# Supplementary material for: Clinical, Radiographic, and Biomechanical Evaluation of the Upper Extremity in Patients with Osteogenesis Imperfecta
Source: J Clin Med. 2024 Aug 31;13(17):5174. doi: 10.3390/jcm13175174 (PMC11396301; doi:10.3390/jcm13175174)
Supplement: Supplementary file 1 [file jcm-13-05174-s001.zip › jcm-3134479-supplementary.pdf]

Table S1. Results of aRoM testing for all examined joints.

	shoulder sag. [°]	shoulder front. [°]	shoulder trans. [°]	elbow sag. [°]	elbow trans. [°]	wrist sag. [°]	wrist front. [°]
**OI mean**	166,2	140,7	106,9	136,9	85,5	144,7	60,7
**OI median**	166,4	137,5	108,2	140,9	86,6	147,7	60,9
**OI std**	20,4	17,6	26,7	11,9	22	22,5	6,6
**OI min**	113,9	95	36,3	103,4	33	101,6	43,2
**OI max**	204,2	108,2	179,4	156,8	128,6	181,5	70,8
**HS mean**	161,9	137,6	117,3	141,7	113,9	142,3	57,8
**HS median**	160	138,6	113,5	140	114	143,5	56,9
**HS std**	12	10,8	20,4	8,9	16,7	18,8	6,4
**HS min**	146	108,5	87,8	127,3	82,9	88,9	47,8
**HS max**	192,6	154,4	156,1	160,6	139	175,2	69,5
**OI mean outside HS mean ± 1*HS std**	**no**	**no**	**no**	**no**	**yes**	**no**	**no**
**OI mean outside HS mean ± 2*HS std**	**no**	**no**	**no**	**no**	**no**	**no**	**no**

OI: Osteogenesis Imperfecta affected subjects (patient group); HS: healthy subjects (control group); std: 1*standard deviation, min: minimum value; max: maximum value.
